# Liquid crystal elastomer shell actuators with negative order parameter

**DOI:** 10.1126/sciadv.aaw2476

**Published:** 2019-04-12

**Authors:** V. S. R. Jampani, R. H. Volpe, K. Reguengo de Sousa, J. Ferreira Machado, C. M. Yakacki, J. P. F. Lagerwall

**Affiliations:** 1 Physics and Materials Science Research Unit, University of Luxembourg, Luxembourg, Grand Duchy of Luxemborg.; 2 Department of Mechanical Engineering, University of Colorado Denver, Denver, CO, USA.

## Abstract

Liquid crystals (LCs) are nonsolids with long-range orientational order, described by a scalar order parameter $\langle P_{2}\rangle =\frac{1}{2}\langle 3{\text{cos}}^{2}\beta -1\rangle$. Despite the vast set of existing LC materials, one-third of the order parameter value range, ^−1^/_2_ < 〈*P*_2_〉 < 0, has until now been inaccessible. Here, we present the first material with negative LC order parameter in its ground state, in the form of elastomeric shells. The optical and actuation characteristics are opposite to those of conventional LC elastomers (LCEs). This novel class of anti-ordered elastomers gives access to the previously secluded range of liquid crystallinity with 〈*P*_2_〉 < 0, providing new challenges for soft matter physics and adding a complementary type of LCE actuator that is attractive for applications in, e.g., soft robotics.

## Teaser

A theoretically predicted case of anti-ordered liquid crystal rubber is realized in the form of spherical artificial muscles.

## INTRODUCTION

The type of order in a physical system is a fundamental quality to define the symmetry (*1*, *2*), functionality (*3*), and responsiveness of materials (*4*). When the order is long range, we can trace the macroscopic optical and mechanical behavior down to the arrangement of the individual constituent molecules. For example, liquid crystals (LCs) are nonsolid condensed phases with long-range orientational order, giving them anisotropic physical properties such as optical birefringence. In conventional LCs, the constituents, referred to as mesogens (they can be individual molecules, aggregates such as micelles, or nanoparticles), align with their principal axis along a common symmetry axis, dubbed the director, **n**. The most commonly studied and technologically most important LC is the nematic phase, exhibiting no other long-range order than orientational. Studied between crossed polarizers, a nematic formed by rod-shaped mesogens with maximum electronic polarizability along the long axis shows positive uniaxial birefringence with **n** as its optic axis, i.e., the refractive index is greater along **n** than perpendicular to it.

**Fig. 1:**
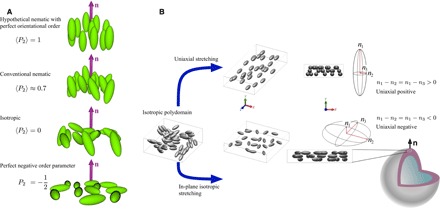
Schematic representation of LCs with varying order parameter. (**A**) Organization of mesogens (represented by ellipsoids) for order parameter varying from 〈*P*_2_〉 = ^−1^/_2_ to 〈*P*_2_〉 = +1. In the isotropic phase, **n** is still required as a reference direction for calculating 〈*P*_2_〉, but its orientation is arbitrary. (**B**) Comparison of the conventional way of making LCEs with uniaxial stretching (top), leading to 〈*P*_2_〉 > 0 and birefringence Δ*n* > 0 (assuming rod-shaped mesogens), and our in-plane isotropic stretching (bottom), yielding LCE shells with 〈*P*_2_〉 < 0 and Δ*n* < 0.

The birefringence of an LC is a function of the type and degree of orientational order, quantified by the nematic order parameter. In three dimensions, it takes the form of the averaged second Legendre polynomial of cos β, $\langle P_{2}(\text{cos}\beta )\rangle =\frac{1}{2}\langle 3{\text{cos}}^{2}\beta -1\rangle$, where β is the angle between an individual mesogen symmetry axis and **n**, and 〈〉 symbolize an ensemble average. The full range is $-\frac{1}{2}<\langle P_{2}\rangle <+1$, but most often, only the positive regime is considered. For an isotropic state, 〈*P*_2_〉 = 0, reflecting no orientational order, whereas a hypothetical perfectly ordered material would be described by 〈*P*_2_〉 = 1 (see Fig. 1A). Conventional LCs typically have 0.3 < 〈*P*_2_〉 < 0.8.

No liquid crystalline system has been discovered that spontaneously develops negative orientational order, 〈*P*_2_〉 < 0, to the best of our knowledge. The Landau-de Gennes theory for the isotropic-nematic transition (*2*) actually predicts a local free energy minimum for 〈*P*_2_〉 < 0, but it is much shallower than the global energy minimum at 〈*P*_2_〉 > 0, explaining the elusive character of the negative order parameter state. This would correspond to a situation where the mesogens avoid a certain direction rather than align along it. They would tend to orient within a plane perpendicular to **n**, without any preferred orientation within that plane (see the bottom schematic of Fig. 1A). The director remains the principal symmetry axis of the phase, thus also of its physical properties. Note that the order parameter is defined on a microscopic scale, i.e., it refers to mesogen orientations compared to **n**, within a volume in which the variation of **n** is negligible. Thus, cases of degenerate in-plane director orientation, achieved, e.g., by subjecting a nematic with negative dielectric anisotropy to an electric field, do not constitute examples of negative LC order parameter. Locally, the mesogens still align along **n** and thus 〈*P*_2_〉 > 0.

Negative order parameter has been used to describe the orientational distribution of gold nanorods dispersed as guests in an LC host phase (*5*, *6*), but this is a different situation. These nanorods did not form an LC phase themselves, but their interactions with the micelles of the host favored local perpendicular arrangement. The actual LC phase was of conventional type with 〈*P*_2_〉 > 0. Very recently, negative order parameter was reported as a transitory state for a uniaxially aligned conventional 〈*P*_2_〉 > 0 liquid crystal elastomer (LCE) that was strongly stretched perpendicular to **n** (*7*, *8*). This was an externally imposed unstable configuration of the LCE existing only as a borderline state where the original director is being reoriented. A similar situation of unstable small negative order parameter induced under compression in isotropic polydimethylsiloxane was reported by Lorthioir *et al*. (*9*).

In our study, we present the first example of a liquid crystalline system that exhibits 〈*P*_2_〉 < 0 for the actual mesogens in the absence of external forces. This is thus the ground state of our system, i.e., it represents the lowest-energy accessible state. As a consequence, the relaxed properties and the responsiveness to external stimuli are fundamentally changed compared to conventional LCEs. We achieve this main-chain LCE with anti-ordered mesogens in the ground state by engineering it mechanically in an unconventional manner, in the form of spherical shells using a bottom-up microfluidic and osmotic stretching approach (Fig. 1B).

LCEs result when chemically reactive mesogens are polymerized into a loosely cross-linked network with low glass transition temperature (*4*, *10*–*13*). If the mesogens are part of the linear polymer chain, as in our case, then we have a main-chain LCE, whereas a side-chain LCE has mesogens attached as pendants to a nonmesogenic polymer backbone. Both of these classes of elastomers (rubbers) with LC order can exhibit spectacular responsiveness, since the orientational order acts on the elastomeric network like an externally applied stretching force. The average polymer conformation is thus not isotropic in the ground state, in contrast to conventional rubbers, with a consequent entropic penalty. For a regular 〈*P*_2_〉 > 0 state, the polymer network of a main-chain LCE is stretched along **n**. If the system is heated to temperatures where the LCE turns isotropic (or approaches an isotropic state), then the effective stretching is released and the conformation changes to isotropic to maximize entropy. The molecular scale adjustment is amplified to a macroscopic shape change, with contraction along **n** and expansion in the perpendicular plane if the ground state had 〈*P*_2_〉 > 0. This actuation mechanism, which is reversible over many cycles because of chemical cross-linking, has been demonstrated in different systems with impressive results (*4*, *14*–*22*), and LCEs are therefore receiving attention in materials science, for instance, as components for soft robotics (*23*–*27*).

## RESULTS

### Synthesis of negative order parameter LCE shells

A popular way of making LCEs with **n** uniform across the sample, initially developed by Finkelmann and Wermter (*27*), combines two steps of polymerization and cross-linking. First, a sparse isotropic network is formed that is mechanically stretched along a selected direction (see Fig. 1B, top scenario). While stretching, the second cross-linking step is done to render the uniaxially ordered state with 〈*P*_2_〉 > 0 permanent. In our work, we use a related two-step strategy to make spherical shells (*28*), surrounded by and containing aqueous isotropic phases, of LCE with 〈*P*_2_〉 < 0. Self-closing spherical LCE actuators are expected to exhibit exotic actuation modes (*29*), and the two cases, so far, where LCE shells were realized (*30*, *31*) (in both cases with 〈*P*_2_〉 > 0) confirmed novel types of motion, giving rise to pumping/suction and buckling, respectively.

We produce our shells in the liquid state, surrounded by immiscible aqueous phases containing glycerol and polyvinyl alcohol (PVA), using a capillary microfluidic process (*32*, *33*). The shell fluid is composed of the (solvent-dissolved) LCE precursors—an LC monomer with two reactive ends (RM257), a linear dithiol [1,6-hexanedithiol (HDT)], a tetrathiol cross-linker [pentaerythritol tetrakis (3-mercaptopropionate) (PETMP)]—and a photoinitiator (see Materials and Methods for details). Since the LCE precursors stay disordered for a long time because of the solvent, the first polymerization step [thiol-acrylate click reaction; see fig. S1 and (*17*, *34*–*36*)] creates an initial sparse network with an architecture that is isotropic in three dimensions. This polymerization is initiated by replacing the original outer phase by a 1 mM solution of the catalyst triethylamine in pure water. Because its solute content is lower than the aqueous phase inside the shell, an osmotic pressure is set up that drives water through the shell into the inner droplet, which thus expands (*37*, *38*).

This process leads to two important outcomes. First, the shells expand in diameter and their walls decrease in thickness because of a Poisson’s effect. The resulting peculiar stretching is key to the behavior of our LCE shells. Because this stretching of the initial network is isotropic in the shell plane, all mesogen directions perpendicular to the shell radius are promoted, yielding the elusive 〈*P*_2_〉 < 0 state. The shell radius becomes the local symmetry axis, i.e., the director **n** (see Fig. 1B, bottom scenario).

The second outcome is that the organic solvent in the shell is removed through gradual evaporation via the aqueous outer phase, leaving the shell in a nematic state. We tuned the precursor mixture composition such that some unreacted RM257 acrylate tails remain after the first polymerization; hence, the final cross-linking step can now be initiated by ultraviolet (UV) irradiation. Unless otherwise mentioned, all shells are photocross-linked at 35°C. This finalizes the LCE structure with a second network that makes the osmotic stretching–induced 〈*P*_2_〉 < 0 situation permanent.

### Optical characterization of 〈*P*_2_〉 < 0 ground-state LCEs

**Fig. 2:**
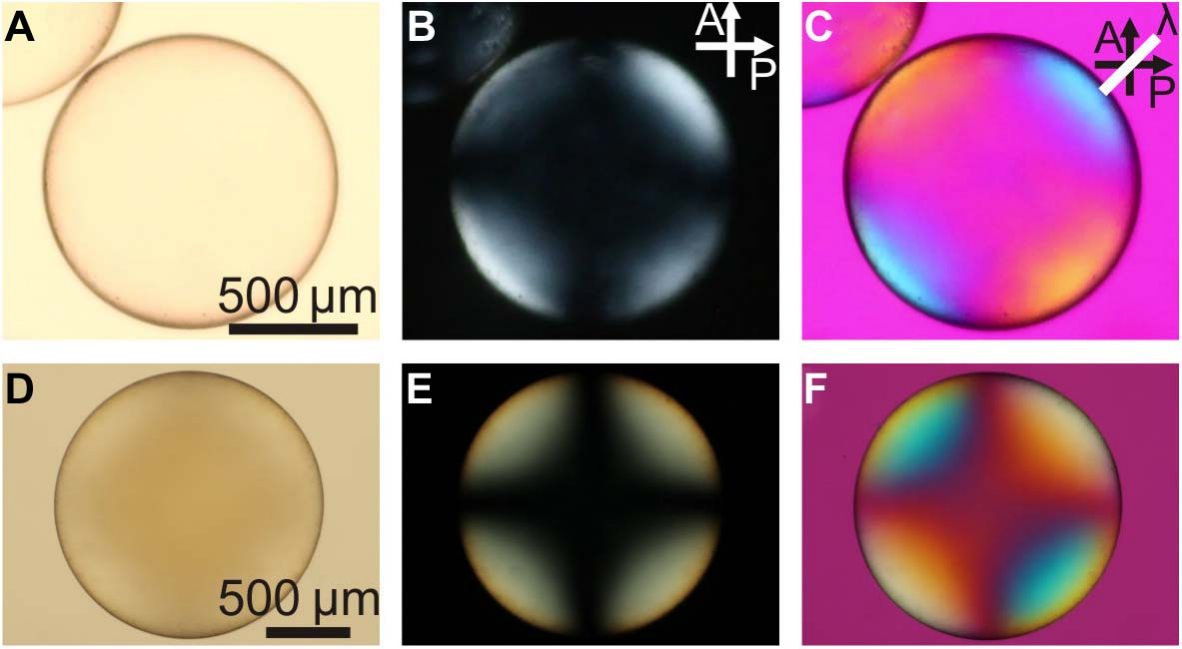
POM images of radially aligned LCE shells with positive (A to C) and negative (D to F) order parameter. The top row shows a shell with Δ*n* > 0 (and 〈P_2_〉 > 0) produced with only dithiols in the precursor mixture, while the bottom row shows a shell with Δ*n* < 0 (and 〈P_2_〉 < 0) produced with tetra- and dithiols in the precursor mixture. The shells are observed (A and D) in transmission without analyzer, (B and E) between crossed polarizers (indicated by P and A), and (C and F) between crossed polarizers with a λ plate inserted (its optic axis is indicated with a white line). In C, the upper left and lower right corners shift to orange, whereas the upper right and lower left corners shift to blue, confirming Δ*n* > 0. In F this color contrast is reversed: the upper left and lower right corners shift to blue, whereas the upper right and lower left corners shift to orange. This confirms that the shell in D to F has Δ*n* < 0.

We investigated the cross-linked shells prepared without (Fig. 2, A to C) and with (Fig. 2, D to F) PETMP cross-linker, respectively, using polarized optical microscopy (POM) in transmission mode. Without analyzer (Fig. 2, A and D) and between crossed polarizers (Fig. 2, B and E), the textures of the two shell types are qualitatively indistinguishable. Both types show the characteristic texture of shells with **n** along the shell radius (*39*). The shells are black at the center, where the light passes along the optic axis, while they appear increasingly brighter toward the corners of the images; the projection of the optic axis into the image plane increases toward the shell perimeter, with ±45° to the polarizer along these directions.

When a λ plate is inserted into the microscope, the textures appear distinctly different for the two shell types. The shell centers now have the first-order magenta color of the λ plate, corresponding to a 530-nm optical path difference. The shell without PETMP (Fig. 2C) shows the behavior of a radially aligned shell with positive uniaxial birefringence, Δ*n* > 0 (*31*). The color shifts to blue—rightward in the Michel-Lévy diagram, thus increasing path difference—in the upper right and lower left corners, while the upper left and lower right corners shift to yellow—leftward in the Michel-Lévy diagram, thus, decreasing path difference. The birefringence effects of shell and λ plate act additively in the upper right and lower left corners, subtractively in the others.

In contrast, the shells prepared with PETMP show the opposite color pattern (see Fig. 2F): The combined birefringence of λ plate and shell is now additive toward the upper left and lower right but subtractive toward the upper right and lower left. This observation conveys two important pieces of information: (i) the birefringence of shells prepared with PETMP is negative uniaxial, Δ*n* < 0, since we know from the textural pattern that **n** is radial within the shell and the optic axis of the λ plate is unchanged. (ii) The mesogen order parameter is negative, 〈*P*_2_〉 < 0, since the mesogens are the same rod-shaped RM257 molecules, with maximum polarizability along the mesogen long axis. The 〈*P*_2_〉 < 0 situation is programmed during the first polymerization step, by the presence of the PETMP cross-linker, when the catalyst is added.

### Thermal response of 〈*P*_2_〉 < 0 LCE shells

Because 〈*P*_2_〉 < 0 implies that the polymer is stretched out perpendicular to **n** in the ground state, rather than along **n** as in conventional main-chain LCEs, mechanical actuation upon temperature change is expected to be reversed (see fig. S3): As the sample is heated and 〈*P*_2_〉 approaches zero from below, the LCE should expand along **n** and contract in the plane perpendicular to **n**. Optically, the effect of heating should be a reduction in the absolute birefringence, |Δ*n*|, as in conventional LCEs because, in both cases, heating yields a reduction in the absolute value of the order parameter, |〈*P*_2_〉|.

When heating a 〈*P*_2_〉 < 0 shell cross-linked at 35° to 75°C (movie S1 and fig. S4), we see essentially no change in the shape or size of the shell. It remains spherical and the radius is unchanged, as highlighted by the identical dashed circles drawn in fig. S4 (A and D). We do see the expected decrease in |Δ*n*|, but it is small. The lack of actuation and weak optical response can be understood as a result of the inability of the spherical shell to reduce its surface area, which is the natural mechanical response to heating of the radially oriented 〈*P*_2_〉 < 0 LCE (fig. S3), while maintaining constant volume as imposed by the incompressible core fluid. However, if we remove the constraint of constant inner volume by cutting a small opening (movie S2 and figs. S5 and S6), we find ~10% reduction in diameter upon heating from 70° to 85°C, equivalent to ~27% reduction of the inner volume.

**Fig. 3:**
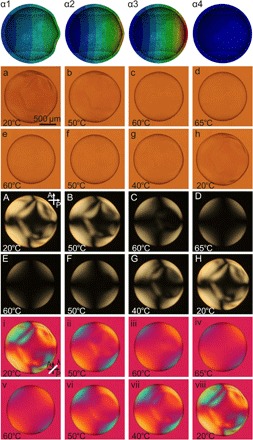
Simulation and transmission POM investigation of thermal response of 〈*P*_2_〉 < 0 LCE shell cross-linked at 60°C. The shell is spherical only at high temperature where it has been cross-linked, whereas it wrinkles and buckles upon cooling, to expand the surface area as 〈*P*_2_〉 gets increasingly negative. Upon heating, the shell contracts in area and expands in thickness, as expected for 〈*P*_2_〉 < 0 approaching 〈*P*_2_〉 = 0. (α**1** to α**4**) Simulation images show stress-induced buckled regions when the inner volume is slightly offset from the center. (**a** to **h**) POM images without analyzer, (**A** to **H**) between crossed polarizers, and (**i** to **viii**) between crossed polarizers with λ plate inserted (optic axis indicated by white line).

While an intact shell cannot decrease its surface area, because of the constant inner volume constraint, it could increase its surface area, which is enabled via buckling. We simulated such a situation (details in the Supplementary Materials), revealing that buckles develop primarily around the thinnest point of a shell (see Fig. 3, top row). To realize this situation experimentally, we modify the shell preparation procedure such that the final cross-linking is performed at 60°C. Now, we have fixed an LCE shell with lowered |〈*P*_2_〉|, but still with 〈*P*_2_〉 < 0 ground state, into a smooth spherical shape. If we cool this LCE to room temperature, the increasing equilibrium |〈*P*_2_〉| (here corresponding to decreasing 〈*P*_2_〉) calls for an increase in surface area. This is what we find, as shown in Fig. 3. For consistency with figs. S4 and S5, we show the shell first during heating from room temperature to 65°C and then cooling back. The room temperature state is now strongly buckled and partially collapsed (Fig. 3, a and h). Upon heating to the original cross-linking temperature, the reduction in surface area as |〈*P*_2_〉| decreases tightens up the shell into a smaller, smooth sphere (Fig. 3, d and e).

**Fig. 4:**
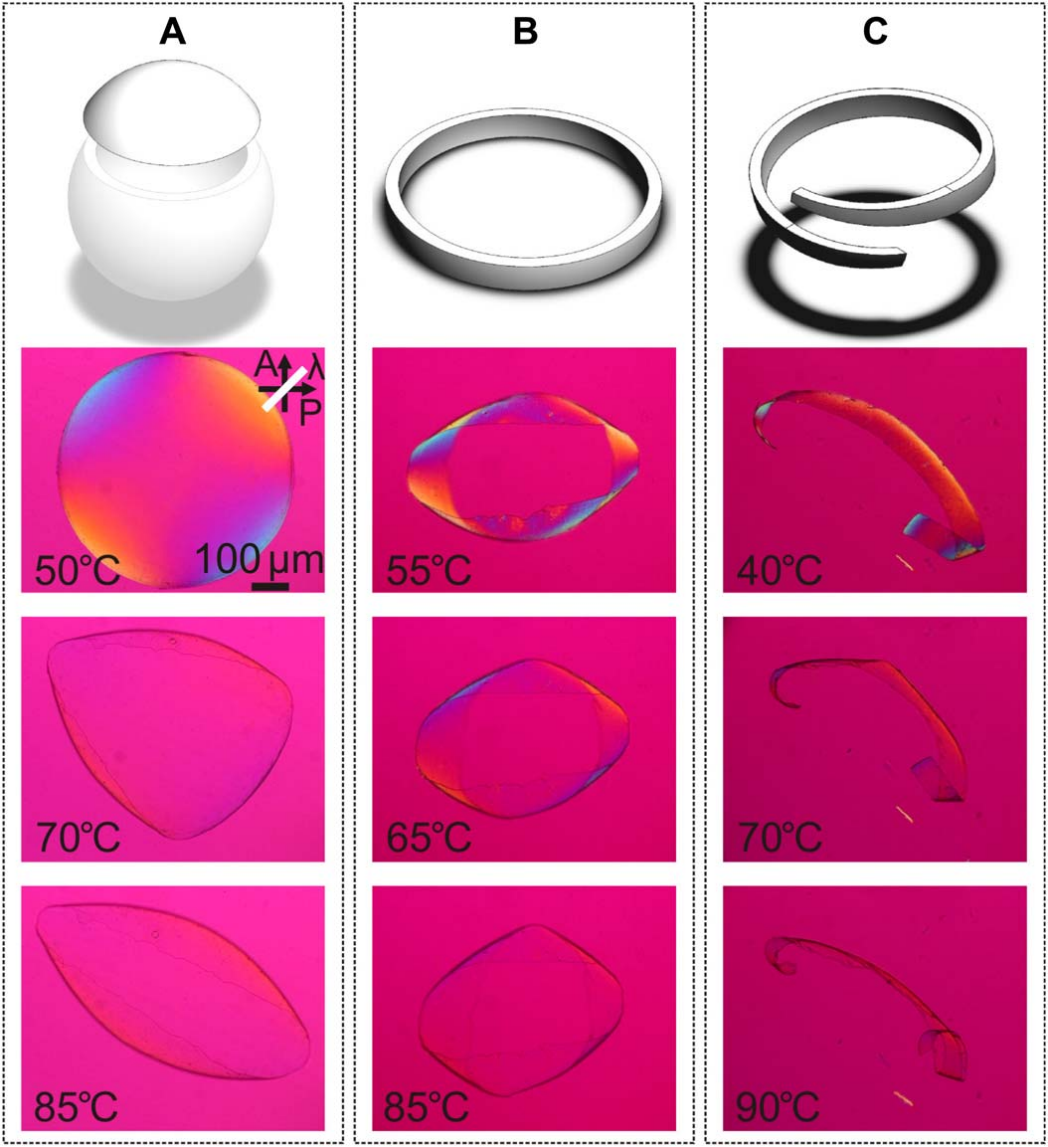
Schematics of 〈*P*_2_〉 < 0 shell fragments and POM investigation of thermal response. (**A**) The shell fragment is close to a spherical cap shape in the ground state, gradually transforming into an ellipsoid upon heating. (**B**) A self-closing ribbon cut near the equator folds and twists upon heating. (**C**) A nonclosing spiral-shaped ribbon fragment shows strong curling and twisting when heated. In all three cases, Δ*n* decreases strongly on heating, approaching zero, in correlation with the shape morphing. A λ plate is inserted during all experiments (optic axis indicated by white line). Movie S5 shows the full actuation cycle for each fragment.

Very interesting motion arises when we subject fragments cut from a shell to temperature changes. As shown for samples placed on beds of glycerol in Fig. 4 and the Supplementary Materials, these fragments exhibit strong responses, tunable by the location and path of cut. If a spherical cap is cut off (Fig. 4A), the shape changes upon actuation (movie S5, beginning), first to triangular and then to a cylindrically rolled-up cap. If instead a self-closing strip is cut along the equator, a flattening-twisting motion can be seen (Fig. 4B and movie S5, middle). Last, if an open-ended spiral is cut, this makes a strong twist and curl at the end of the actuation process (Fig. 4C and movie S5, end). All actuation modes are fully reversible upon cooling, and in all cases, the fragments lose almost all birefringence in the actuated state, indicating that they acquire 〈*P*_2_〉 ≈ 0 at 85° to 90°C.

**Fig. 5:**
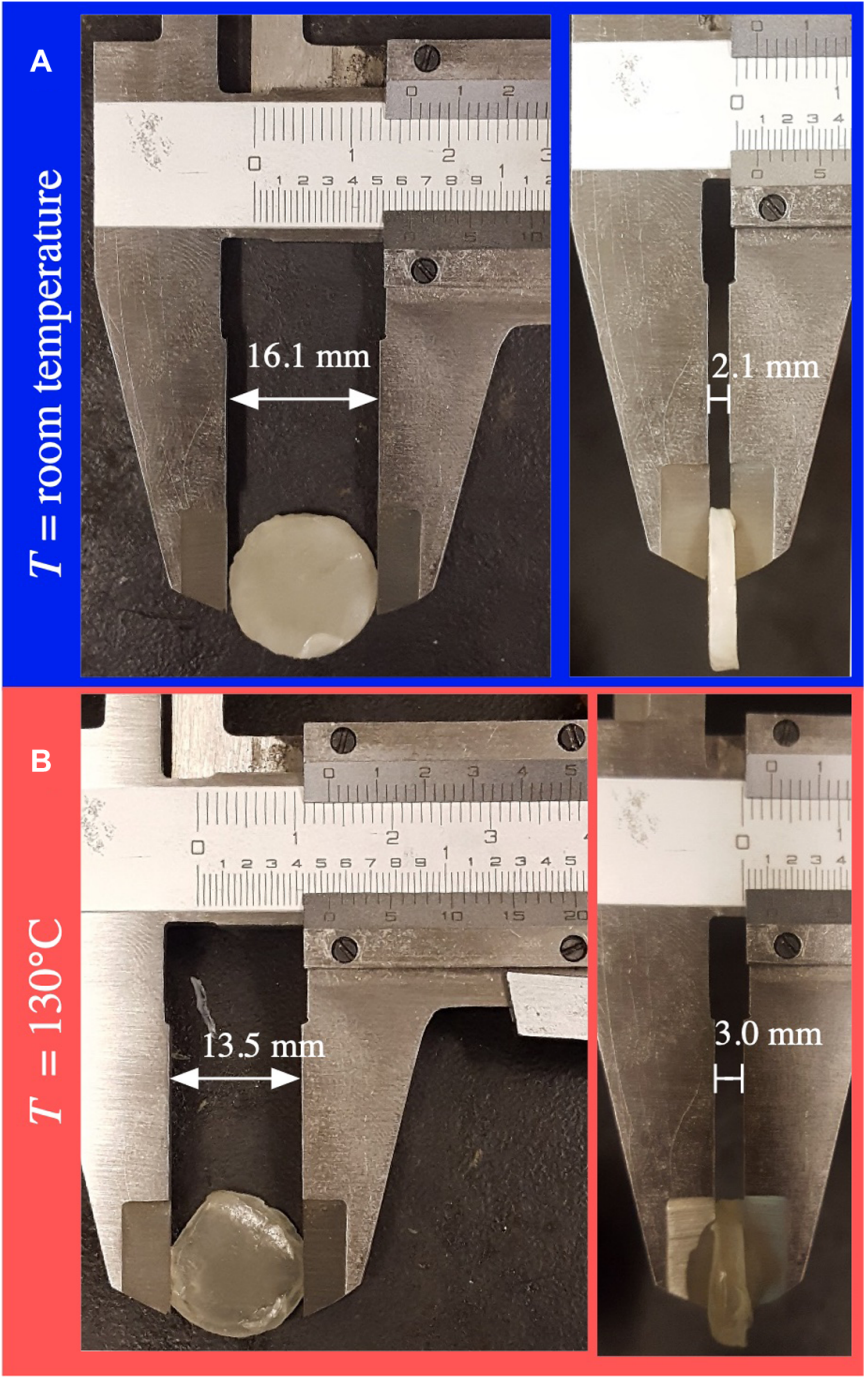
Actuation of macroscopic 〈*P*_2_〉 < 0 disk. (**A**) The cylindrical disk, which was compressed along its symmetry axis between the two polymerization stages, is measured at room temperature before actuation. (**B**) Above the clearing point, at 130°C, the disk is markedly thicker, while it has contracted in the disk plane, opposite to the behavior of a 〈*P*_2_〉 > 0 LCE.

The approach realized through the shell production for achieving main-chain LCEs with 〈*P*_2_〉 < 0 should be realizable also in other geometries and at a larger scale. The isotropic in-plane stretching due to osmosis is mechanically similar to uniaxial compression; hence, we should expect the same type of order if we compress along a single direction the sparse isotropic network formed after the first-stage polymerization. This is what happens, as illustrated in Fig. 5, for a macroscopic disk that was compressed to 41% of its original thickness (from 5.1 to 2.1 mm) between the two stages of polymerization (manufacturing details are found in the Supplementary Materials). Along **n**, as defined by the compression axis, the disk expands upon heating to 130°C, from 2.1 to 3.0 mm (43% thickness expansion), and in the perpendicular plane, it contracts from 16.1 to 13.5 mm in diameter (30% area contraction), demonstrating that the disk has 〈*P*_2_〉 < 0. The lack of volume conservation is most likely due to the imperfect control of the director orientation throughout the macroscopic disk, which also gives it an opaque optical character, in the ground state and at high temperature. This unfortunately precludes a corresponding optical characterization. This experiment serves to demonstrate that the concept can be scaled up and applied to other geometries than shells. With further refinement, we are confident that the control of the macroscopic orientation can be considerably improved.

## DISCUSSION

The connection between Δ*n* and 〈*P*_2_〉 can be formalized by writing the birefringence Δ*n* ∝ α_∥_ − α_⊥_, with α_∥_ and α_⊥_ the effective mesogen polarizabilities along and perpendicular to the director **n**, respectively (*40*). In the case of rod-like mesogens, α_∥_ = 〈*P*_2_〉α_*l*_ and α_⊥_ = 〈*P*_2_〉α_*s*_, where α_*l*_ is the polarizability along the mesogen long axis and α_*s*_ is the polarizability along the short axis. Since our rod-shaped mesogens have Δα = α_*l*_ − α_*s*_ > 0, they can give rise to Δ*n* < 0 only if they organize in an arrangement with 〈*P*_2_〉 < 0. Because of the curved geometry of our shells, with radial optic axis, it is very difficult to quantitatively measure 〈*P*_2_〉. However, the fact that Δ*n* is comparable for the two shells in Fig. 2 indicates that the degree of negative ordering in the lower shell is comparable to that of positive ordering in the upper shell. We can thus estimate 〈*P*_2_〉 to be on the order of at least −0.1, i.e., the negative order parameter is substantial. We are developing a methodology for reliable quantitative measurement of the negative 〈*P*_2_〉 and will publish the results separately.

The fact that buckles appear on cooling below the cross-linking temperature (Fig. 3) is noteworthy, as it indicates that the network formed during osmotic stretching does not define the ground state; it only introduces a strong kinetic barrier against reaching the global LC phase energy minimum at 〈*P*_2_〉 > 0 (*2*). Upon cooling below the cross-linking temperature, the system therefore goes deeper into the secondary minimum at 〈*P*_2_〉 < 0, leading to stronger negative order parameter than what was induced by stretching. It is also notable that the response upon cooling here is comparable to that observed by Jampani *et al*. (*31*) for a radially aligned shell with 〈*P*_2_〉 > 0 upon heating. The reduction of 〈*P*_2_〉 toward zero upon heating of the conventional LCE shell generated expansion of the shell area, triggering buckling, rather than contraction upon heating as in the present case (Fig. 3). This is a clear testimony to the 〈*P*_2_〉 < 0 ground state and its inverting effect on actuation.

Apart from the curvature of our shells, the situation in our LCE production is mechanically comparable to the “anisotropic deswelling” method introduced by Finkelmann and Kim (*41*) for macroscopically aligning cholesteric side-chain LCEs, as well as to the mechanical compression of side-chain LCE pillars explored by the Sánchez-Ferrer team (*42*). However, because the mesogens were not part of the polymer backbone in these studies, they did not result in negative mesogen orientational order but rather a polydomain arrangement with 〈*P*_2_〉 > 0 and **n** randomly oriented in the plane perpendicular to the deswelling direction. In contrast, by using a main-chain LCE, we achieve 〈*P*_2_〉 < 0 and **n** perpendicular to the stretching plane, along the osmotic expansion direction in the case of the shells.

From an applied point of view, we have realized a new class of LCE actuators that is highly complementary to that of conventional 〈*P*_2_〉 > 0 LCEs. The actuation response (fast) of a 〈*P*_2_〉 < 0 LCE corresponds to the relaxation response (slow) of a 〈*P*_2_〉 > 0 LCE with otherwise identical configuration (*31*); hence, the two could work very effectively in tandem. The change in actuation upon reversing sign of 〈*P*_2_〉 is fundamentally different from the change upon simply reorienting **n**: The unidirectional expansion along **n** upon heating a 〈*P*_2_〉 < 0 LCE cannot be reproduced with 〈*P*_2_〉 > 0 because, in that case, the heating-induced expansion is always across the full plane perpendicular to **n**.

## CONCLUSION

We have realized the first ever LCE with negative mesogen order parameter ground state, 〈*P*_2_〉 < 0, by producing shells of a lightly cross-linked pre-LCE of positive polarizability anisotropy molecules, and we have demonstrated transferability of the concept also to large samples of different shape. Our procedure provides a convenient tool to reproducibly produce 〈*P*_2_〉 < 0 LCEs, opening the door to a previously unexplored domain of LC and soft matter research. A relevant future study will be to systematically study the order parameter quantitatively as a function of uniaxial compression/in-plane isotropic stretching between the two polymerization stages. Moreover, by incorporating azo dyes, it should be possible to isothermally drive the 〈*P*_2_〉 < 0 → 〈*P*_2_〉 = 0 actuation by UV light illumination.

## MATERIALS AND METHODS

### Materials, shell production, and polymerization

We used only commercially available materials in our experiments, following the thiol-acrylate Michael addition and photopolymerization approach. As shown in fig. S1, the reaction involves one reactive rod-shaped mesogen with dual acrylate end groups, code-named RM257 (Wilshire Technologies); one linear dithiol, HDT (Sigma-Aldrich); and one cross-shaped tetrathiol, PETMP (Sigma-Aldrich). The former thiol acted as a spacer, and the latter acted as a cross-linker for the initial sparse network. These components were dissolved in dichloromethane (Sigma-Aldrich) together with 10% Irgacure 819 photoinitiator (Ciba). The standard composition of LCE precursor solution had RM257, HDT, and PETMP in a molar ratio of 1:1:0.2. While this always resulted in ground-state negative order parameter shells, 〈*P*_2_〉 < 0, removal of the cross-shaped tetrathiol from the solution led to shells with positive order parameter, 〈*P*_2_〉 > 0. Depending on the targeted shell type, this mixture composition was adjusted, with varying amounts of linear HDT and cross-shaped PETMP. For confocal fluorescence imaging, we dissolved 3 weight % (wt %) dichroic azo dye named DDR536 (Color Synthesis Solutions) into the LCE precursor solution.

Following the principle of Utada *et al*. (*33*), nested square and cylindrical glass capillaries were used for shell production, as shown in fig. S2. The cylindrical injection tube of 1-mm outer diameter was tapered using a pipette puller (P-1000, Sutter Instruments) and cut to an orifice diameter of 90 μm using a microforge (Narishige). The collection tube, also cylindrical with a 1-mm outer diameter, was likewise tapered and cut to an orifice diameter of around 400 μm. Both tapered cylindrical capillaries were inserted into a square capillary of 1-mm inner side length, facing each other, thus creating a flow-focused junction. The interstitial space in the square capillary outside the cylindrical capillaries supported the outer and middle fluid flow, directed against each other. All three fluids met at the junction, in such a way that the middle fluid encapsulated the inner one, the former making a shell around the latter, suspended in the outer fluid. The resulting multiple emulsion was harvested through the collection tube (fig. S2A). The injection capillary was given a hydrophobic surface before assembly using an aqueous solution of 2 wt % fluorinated silane (nonafluorohexyl-triethoxysilane, Gelest). The square capillary and collection tube were treated with 2 wt % methoxytriethyle-neoxypropyltrimethoxysilane (SIM 6493.4, Gelest) in water for ensuring strongly hydrophilic surfaces. We used 5 wt % solutions of PVA (average molar mass, 13 to 18 kg/mol; 87% hydrolyzed) in a water-glycerol mixture of 1:1 volume ratio for inner and outer fluids. Various compositions of LCE precursor solution were used as the middle fluid.

To initiate the first step polymerization, we added 1 mM aqueous solutions of the catalyst triethylamine (Sigma-Aldrich) to the outer aqueous phase. Specifically, the outer phase was diluted by replacing 5 ml of it with the same amount of 1 mM aqueous triethylamine solution. This initiated the thiol acrylate reaction while the shell was undergoing osmosis at the same time. We repeated this step every 24 hours for 3 days. At this stage of the experiment, we had an isotropic sparse thiol-acrylate network throughout the shell. Further osmosis reduced the shell wall thickness and stretched the network in the shell plane, thus inducing negative order. The second-stage polymerization was carried out using a UV light-emitting diode (UVATA LED UV system; peak wavelength, 370 nm; full power, 8800 mW cm^−2^), kept at a distance of 10 cm above the sample vial. This step initiated the second polymerization of the unreacted excess acrylate groups; thus the configuration obtained through osmosis and solvent removal processes was locked in place. As the shell contained an unusually large quantity of photoinitiator from the start, extra care was necessary not to expose the shells to light with wavelengths shorter than 550 nm, as this would initiate the second-step photocrosslinking reaction prematurely. The reason for the unusually high concentration of photoinitiator is that much photoinitiator was lost during the osmosis and solvent removal stage, as were, to some extent, the two thiols. Therefore, these were also present at higher mole fractions than would normally be the case for this LCE chemistry.

### Microscope characterization

The shell production was carried out on an inverted microscope (Nikon Eclipse LV 100ND), allowing continuous monitoring of the process. The most important technique for characterizing LCs is POM, as the birefringence of the sample reveals a great deal of information about the way in which the molecules order. Hence, postproduction optical characterization of LCE shells was carried out using mainly POM, here, an upright Olympus BX51 working in transmission mode. The actuation sequence was studied by changing the temperature using a Linkam hot stage (TMS 600, T95-PE). The shell actuation was captured using a color camera (EOS 760D, Canon) mounted on the POM. The optical properties in our shells are dominated by the RM257 component, as this is the only aromatic molecule, thus with large polarizability anisotropy. Its rod-like shape means that the polarizability is highest along the molecule long axis. The first-order phase plate (530 nm) was used to confirm the molecular orientation in LCE shells and a ground-state negative order in LCE shells.

To allow thermal actuation in shells cross-linked at 35°C, we cut small holes in, or cut fragments from, several such shells. To allow clean cuts, the LCE shells were transferred to a petri dish containing pure water, and the petri dish was then cooled to −25°C, turning the inner and outer aqueous media to ice. A scalpel was used for cutting the frozen shell in the desired way, either by cutting a hole or removing a small piece of the shell. For studying thermal actuation, the shell with hole or shell fragment was transferred to a glycerol bath. We have used a heating/cooling rate of 30°C/min in all our experiments in this work.

### Fluorescence confocal microscopy

For 3D visualization of an LCE shell actuation, we used a NIKON A1R+ laser scanning fluorescence confocal system. The fluorescence labeling was achieved by dissolving the dichroic azo dye (DDR 536) into the LCE precursor solution. After the final UV cross-linking stage, the desired shell was transferred to a rectangular glass capillary (inner dimensions, 2 mm by 4 mm; wall thickness, 0.4 mm) filled with pure water for confocal fluorescence measurements. The dye was excited with a 514-nm laser, and the emission was collected in the spectral region of 550 to 600 nm.

### Simulation of shell actuation and comparison with experiment

LCE shells were modeled using ABAQUS finite element software (SIMULIA, Johnston, RI, USA). Actuation was modeled as a thermal expansion (α = −0.002) in the tangential directions around the shell and a contraction in the thickness (α = 0.003). Material properties of the shell were assumed to be isotropic with a modulus of 150 MPa and a Poisson ratio of 0.48. A temperature change was applied to the model to induce approximately a 4 to 5.2% biaxial strain along the shell surface. The inner surface was assigned a fluid cavity interaction with a fluid density of 1 g/cm^3^ to simulate water filling the LCE shell. Two shell configurations were tested: one where the inner cavity shared the same center point as the outer shell to create a perfect sphere, and the other where the centers were offset by 5% of the outer diameter. To ensure modeling accuracy, mesh density was increased until the thinnest portions of the shell were at least two elements in thickness. The perfectly spherical shell was modeled as a 500-μm sphere with a wall thickness of 10 μm, while the offset shell was modeled as an outer sphere and a center cavity with its center offset by 5% of the outer diameter and a wall thickness of 10 μm at the thinnest portion of the shell.

## Supplementary Material

http://advances.sciencemag.org/cgi/content/full/5/4/eaaw2476/DC1

Download PDF

Movie S1

Movie S2

Movie S3

Movie S4

Movie S5

## SUPPLEMENTARY MATERIALS

Supplementary material for this article is available at http://advances.sciencemag.org/cgi/content/full/5/4/eaaw2476/DC1 Fig. S1. LCE precursor molecules and reaction mechanism for conventional 〈*P*_2_〉 > 0 LCEs. Fig. S2. Schematic representation and micrograph of actual shell production. Fig. S3. Schematic representation of actuation in radial shells of LCE with negative and positive order parameters. Fig. S4. POM investigation of thermal response of pristine 〈*P*_2_〉 < 0 LCE shell with radial director. Fig. S5. POM investigation of shell actuation. Fig. S6. Fluorescence confocal microscopy images of a shell with an opening. Fig. S7. Transmission POM investigation of thermal response of 〈*P*_2_〉 < 0 LCE shell fragment. Fig. S8. Fluorescence confocal microscopy images of a shell fragment. Fig. S9. LCE shells were modeled using ABAQUS finite element software. Fig. S10. LCE shell fragment in the shape of twisted ribbon. Fig. S11. Macroscopic disk with negative mesogen order parameter. Movie S1. The video shows thermal actuation cycles (25° to 75°C) of a pristine shell UV cross-linked at 35°C, recorded with a first-order waveplate, crossed polarizers, and one polarizer in the light path, respectively. Movie S2. Thermal actuation of a shell UV cross-linked at 35°C with a hole at the top, immersed in glycerol. Movie S3. The video shows actuation of a fragment cut from a shell UV cross-linked at 35°C. Movie S4. Thermal actuation of a shell UV cross-linked at 60°C. Movie S5. Complex modes of actuation observed by cutting a shell UV cross-linked at 35°C into different topological objects such as a cap shape, a self-closed ribbon, and a long spiral stripe shape.
