# Supplementary material for: Liquid crystal elastomer shell actuators with negative order parameter
Source: Sci Adv. 2019 Apr 12;5(4):eaaw2476. doi: 10.1126/sciadv.aaw2476 (PMC6461453; doi:10.1126/sciadv.aaw2476)
Supplement: http://advances.sciencemag.org/cgi/content/full/5/4/eaaw2476/DC1 [file supp_5_4_eaaw2476__index.html]

Science Advances | Science Advances

## Supplementary Materials

**The PDF file includes:**

- Fig. S1. LCE precursor molecules and reaction mechanism for conventional 〈*P*2〉 > 0 LCEs.
- Fig. S2. Schematic representation and micrograph of actual shell production.
- Fig. S3. Schematic representation of actuation in radial shells of LCE with negative and positive order parameters.
- Fig. S4. POM investigation of thermal response of pristine 〈*P*2〉 < 0 LCE shell with radial director.
- Fig. S5. POM investigation of shell actuation.
- Fig. S6. Fluorescence confocal microscopy images of a shell with an opening.
- Fig. S7. Transmission POM investigation of thermal response of 〈*P*2〉 < 0 LCE shell fragment.
- Fig. S8. Fluorescence confocal microscopy images of a shell fragment.
- Fig. S9. LCE shells were modeled using ABAQUS finite element software.
- Fig. S10. LCE shell fragment in the shape of twisted ribbon.
- Fig. S11. Macroscopic disk with negative mesogen order parameter.
- Legends for Movies S1 to S5

Download PDF

**Other Supplementary Material for this manuscript includes the following:**

- Movie S1 (.mp4 format). The video shows thermal actuation cycles (25° to 75°C) of a pristine shell UV cross-linked at 35°C, recorded with a first-order waveplate, crossed polarizers, and one polarizer in the light path, respectively.
- Movie S2 (.mp4 format). Thermal actuation of a shell UV cross-linked at 35°C with a hole at the top, immersed in glycerol.
- Movie S3 (.mp4 format). The video shows actuation of a fragment cut from a shell UV cross-linked at 35°C.
- Movie S4 (.mp4 format). Thermal actuation of a shell UV cross-linked at 60°C.
- Movie S5 (.mp4 format). Complex modes of actuation observed by cutting a shell UV cross-linked at 35°C into different topological objects such as a cap shape, a self-closed ribbon, and a long spiral stripe shape.

**Files in this Data Supplement:**

- Adobe PDF - aaw2476\_SM.pdf
