# Supplementary material for: Liquid crystal elastomer shell actuators with negative order parameter
Source: Sci Adv. 2019 Apr 12;5(4):eaaw2476. doi: 10.1126/sciadv.aaw2476 (PMC6461453; doi:10.1126/sciadv.aaw2476)
Supplement: Download PDF [file aaw2476_SM.pdf]

Science Advances
AAAS

advances.sciencemag.org/cgi/content/full/5/4/eaaw2476/DC1

Supplementary Materials for

Liquid crystal elastomer shell actuators with negative order parameter

V. S. R. Jampani, R. H. Volpe, K. Reguengo de Sousa, J. Ferreira Machado, C. M. Yakacki, J. P. F. Lagerwall*

*Corresponding author. Email: jan.lagerwall@lcsoftmatter.com

Published 12 April 2019, *Sci. Adv.* **5**, eaaw2476 (2019)
DOI: 10.1126/sciadv.aaw2476

The PDF file includes:

Fig. S1. LCE precursor molecules and reaction mechanism for conventional $\langle P_2 \rangle > 0$ LCEs.
Fig. S2. Schematic representation and micrograph of actual shell production.
Fig. S3. Schematic representation of actuation in radial shells of LCE with negative and positive order parameters.
Fig. S4. POM investigation of thermal response of pristine $\langle P_2 \rangle < 0$ LCE shell with radial director.
Fig. S5. POM investigation of shell actuation.
Fig. S6. Fluorescence confocal microscopy images of a shell with an opening.
Fig. S7. Transmission POM investigation of thermal response of $\langle P_2 \rangle < 0$ LCE shell fragment.
Fig. S8. Fluorescence confocal microscopy images of a shell fragment.
Fig. S9. LCE shells were modeled using ABAQUS finite element software.
Fig. S10. LCE shell fragment in the shape of twisted ribbon.
Fig. S11. Macroscopic disk with negative mesogen order parameter.
Legends for Movies S1 to S5

Other Supplementary Material for this manuscript includes the following:

(available at advances.sciencemag.org/cgi/content/full/5/4/eaaw2476/DC1)

Movie S1 (.mp4 format). The video shows thermal actuation cycles (25° to 75°C) of a pristine shell UV cross-linked at 35°C, recorded with a first-order waveplate, crossed polarizers, and one polarizer in the light path, respectively.
Movie S2 (.mp4 format). Thermal actuation of a shell UV cross-linked at 35°C with a hole at the top, immersed in glycerol.
Movie S3 (.mp4 format). The video shows actuation of a fragment cut from a shell UV cross-linked at 35°C.
Movie S4 (.mp4 format). Thermal actuation of a shell UV cross-linked at 60°C.

Movie S5 (.mp4 format). Complex modes of actuation observed by cutting a shell UV cross-linked at 35°C into different topological objects such as a cap shape, a self-closed ribbon, and a long spiral stripe shape.

Supplementary Materials:

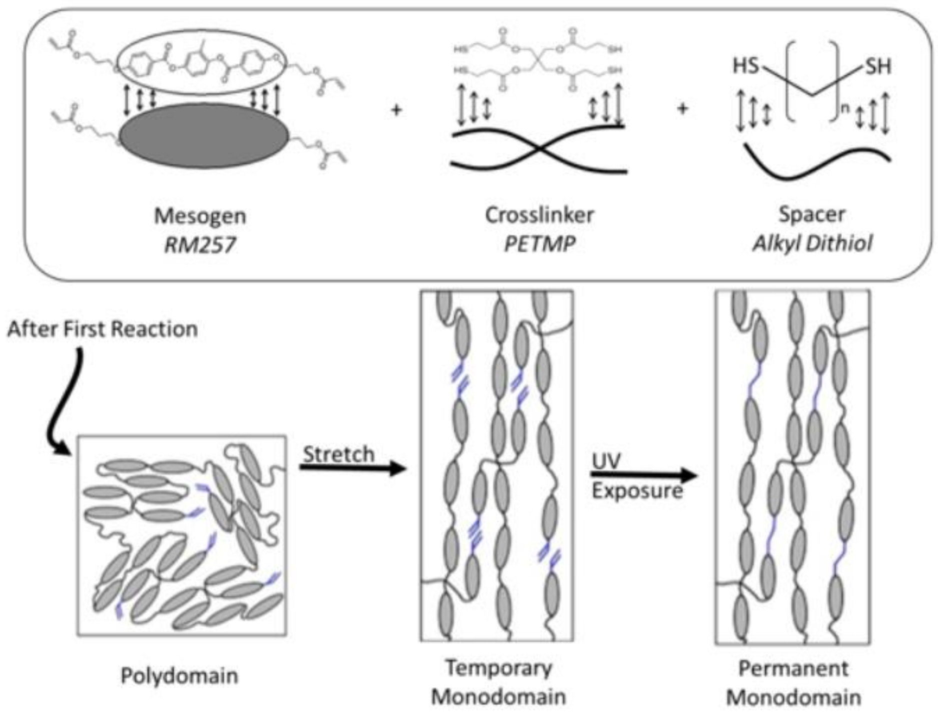

Fig. S1. LCE precursor molecules and reaction mechanism for conventional $\langle P_2 \rangle > 0$ LCEs. Thiol–Acrylate Michael Addition and Photopolymerization (TAMAP) reaction using RM257 diacrylate mesogen, tetra-functional thiol crosslinker, and alkyl dithiol spacer. After reacting these components with a base-catalyst to form a polydomain sparse network, the second stage of the reaction is performed by stretching and applying UV light to the sample. For simplicity, the latter procedure is here illustrated only for the conventional case of uniaxial stretching.

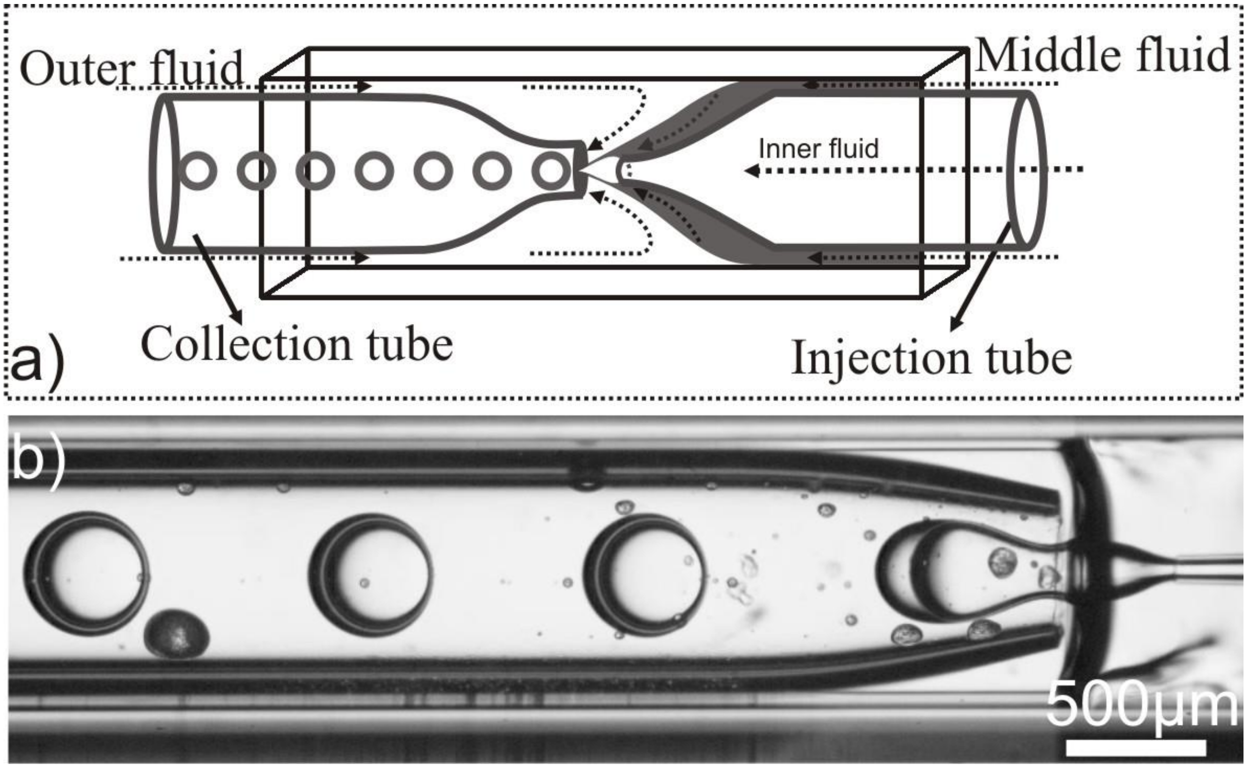

Fig. S2. Schematic representation and micrograph of actual shell production. (a) Cartoon drawing shows flow assisted geometry for the shell production using immiscible fluids. (**b**) A high-speed video snapshot of shell production. The design follows the principle of Utada et al. (*33*).

Expected actuation

Comparing main-chain LCEs with $\langle P_2 \rangle < 0$ and the conventional case of $\langle P_2 \rangle > 0$, we should expect opposite actuation behavior. As the $\langle P_2 \rangle < 0$ LCE is heated and $\langle P_2 \rangle$ approaches zero from below, the LCE should expand along **n** and contract in the plane perpendicular to **n**, as shown from left to middle in fig. S3. In contrast, a conventional LCE contracts along **n** and expands in the perpendicular plane, going from right to middle in fig. S3.

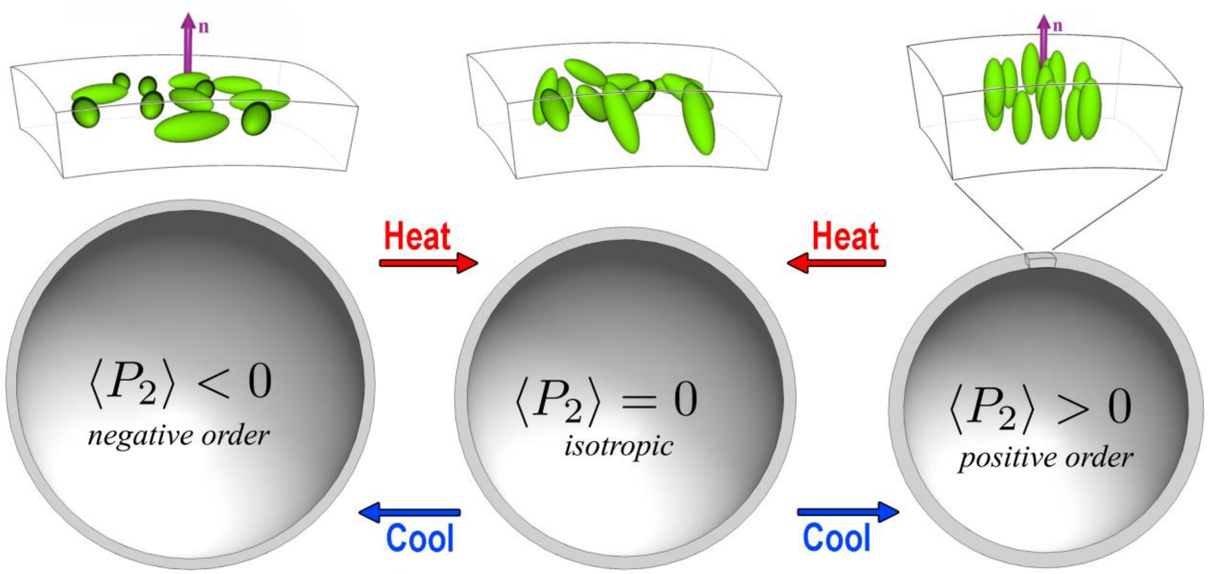

Fig. S3. Schematic representation of actuation in radial shells of LCE with negative and positive order parameters. Heating a shell with $\langle P_2 \rangle < 0$ should lead to shell thickening and reduction of diameter (left to middle), whereas a conventional $\langle P_2 \rangle > 0$ LCE shell should get thinner and expand in diameter (right to middle).

Polarization Optical Microscopy investigations

We studied a $\langle P_2 \rangle < 0$ LCE shell that had been crosslinked at 35°C upon heating it from room temperature to 75°C, see fig. S4. With inserted λ plate (a-h) the central magenta-colored area expands and the colors at the perimeter shift towards red along the optic axis of the λ plate and towards blue in the perpendicular direction. This indicates a reduction in $|\Delta n|$, which is reversible on cooling. It is also recognized without λ plate through an expansion of the black central cross, fig. S4A-H. While these optical observations could have been produced from a thinning of the material, this is ruled out by the absence of any visible shape change.

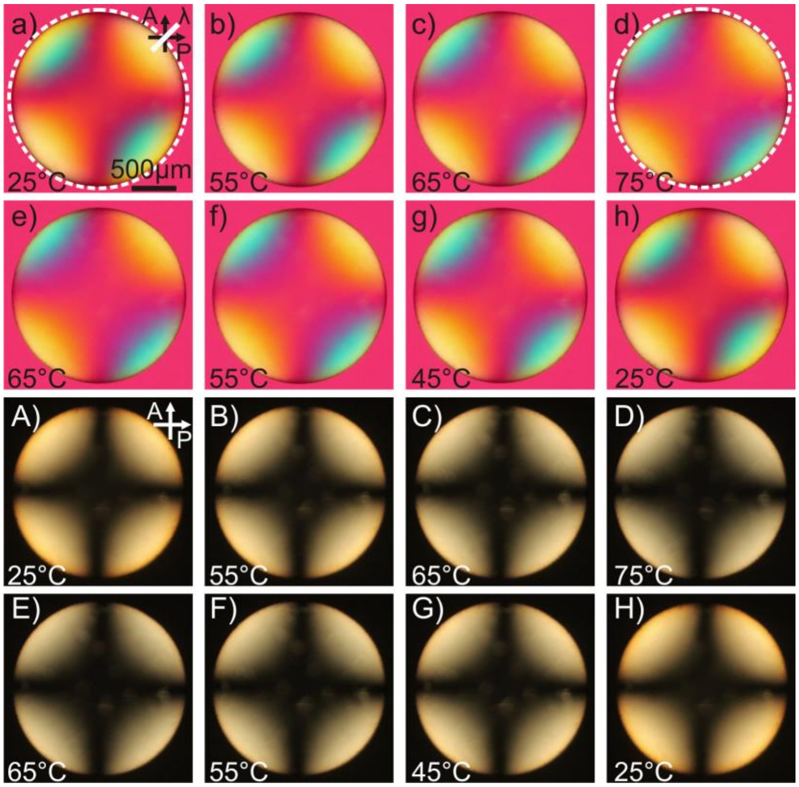

Fig. S4. POM investigation of thermal response of pristine $\langle P_2 \rangle < 0$ LCE shell with radial director. The shell was photocrosslinked at 35°C. Due to the incompressible inner phase, no notable shape change is observed, as highlighted by the identical dashed circles in a and d. A slight change in interference colors reflects a small decrease upon heating of $|\Delta n|$ and of $|\langle P_2 \rangle|$. (a - h) Transmission POM with inserted first-order λ plate (optic axis from lower left to upper right). (**A** to **H**) Equivalent sequence without λ plate; the decrease in $|\Delta n|$ is recognized by the growth in size of the black cross.

The actuation of a shell with a hole is shown in Movie S2 and actuating fragments are shown in Movies S3 and S5. Representative images from the actuation of a shell with a hole are shown in fig. S5. The shell diameter is clearly reduced around 70°C, while it returns to its initial diameter on cooling. There is a thermal hysteresis (fig. S5 (g-i)) due to delay in removing the heat from the glycerol solution in the cooling part of the cycle. The repeatability is demonstrated in images (A-J), obtained with the shell between crossed polarizers, and i-x, where a λ plate has been inserted. Moreover, the polarized light images show the reversible change in birefringence connected to the change in the shell diameter. The image sequence i-x confirms that the shell order parameter remains negative ($\langle P_2 \rangle < 0$) also after cutting the hole. Finally, we have added a sequence of images focused on the shell equator (I - V) rather than the shell opening (a – j, A – J, and i – x), showing that there is approximately 10% reduction in the shell diameter (see also movie S3).

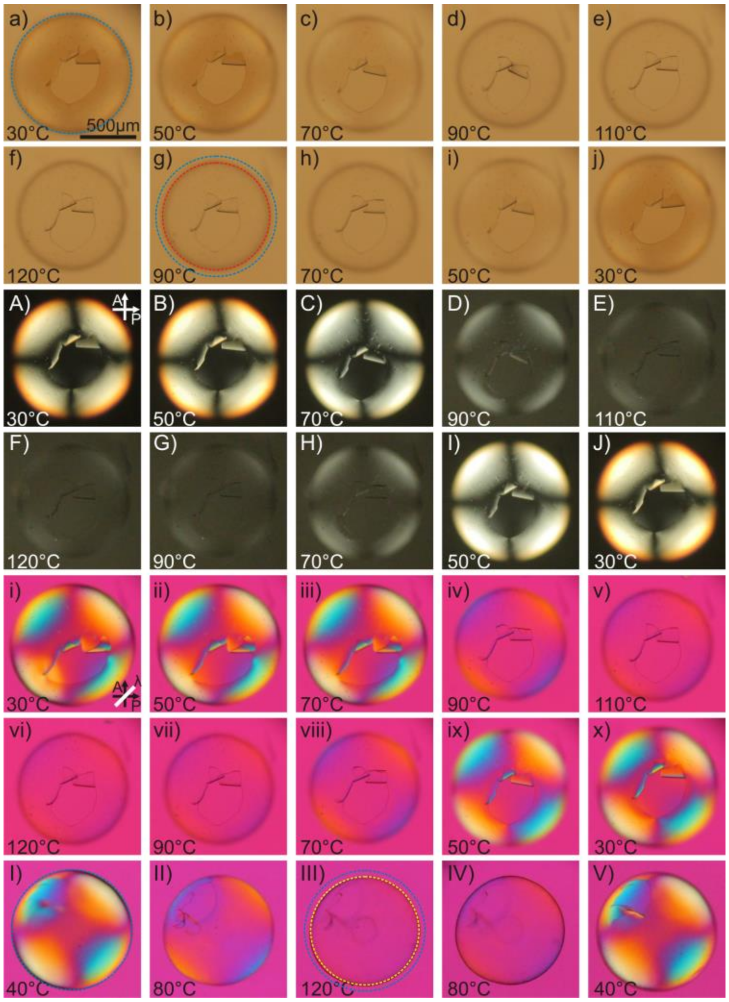

Fig. S5. POM investigation of shell actuation. (a-j) Sequential polarized bright field images of a pierced LCE shell under actuation. The focus plane is on the shell opening. Marked blue and red circles show a substantial decrease in the shell's diameter during the actuation. (**A** to **J**) Crossed polarizer images show a reversible change in birefringence along with a reversible change in the shell diameter. (i-x) First order waveplate images show a gradual decrease in birefringence deduced through the interference color shift to isotropic magenta color. (I-V) Sequential snapshots focused on the shell's equatorial plane taken at different temperatures confirms a change in the shell size.

Fluorescence confocal microscopy

As shown in fig. S6, the shell with a hole on the bottom clearly shows a substantial decrease in its diameter at 75°C compared to the 25°C case. The sequential snapshots of *z*-scans show the fluorescence signal collected at different shell planes. The striking difference in diameter can be seen at the equatorial plane of the shell (z = 400 µm) in panel A and Panel B. The whole 3D shell shape stays spherical during actuation, confirming the observation in fig. S4. The change in diameter accounts to 10%, which is equivalent to change in the internal volume of approximately 27% considering a negligible increase in shell thickness towards inner volume of the shell during the actuation.

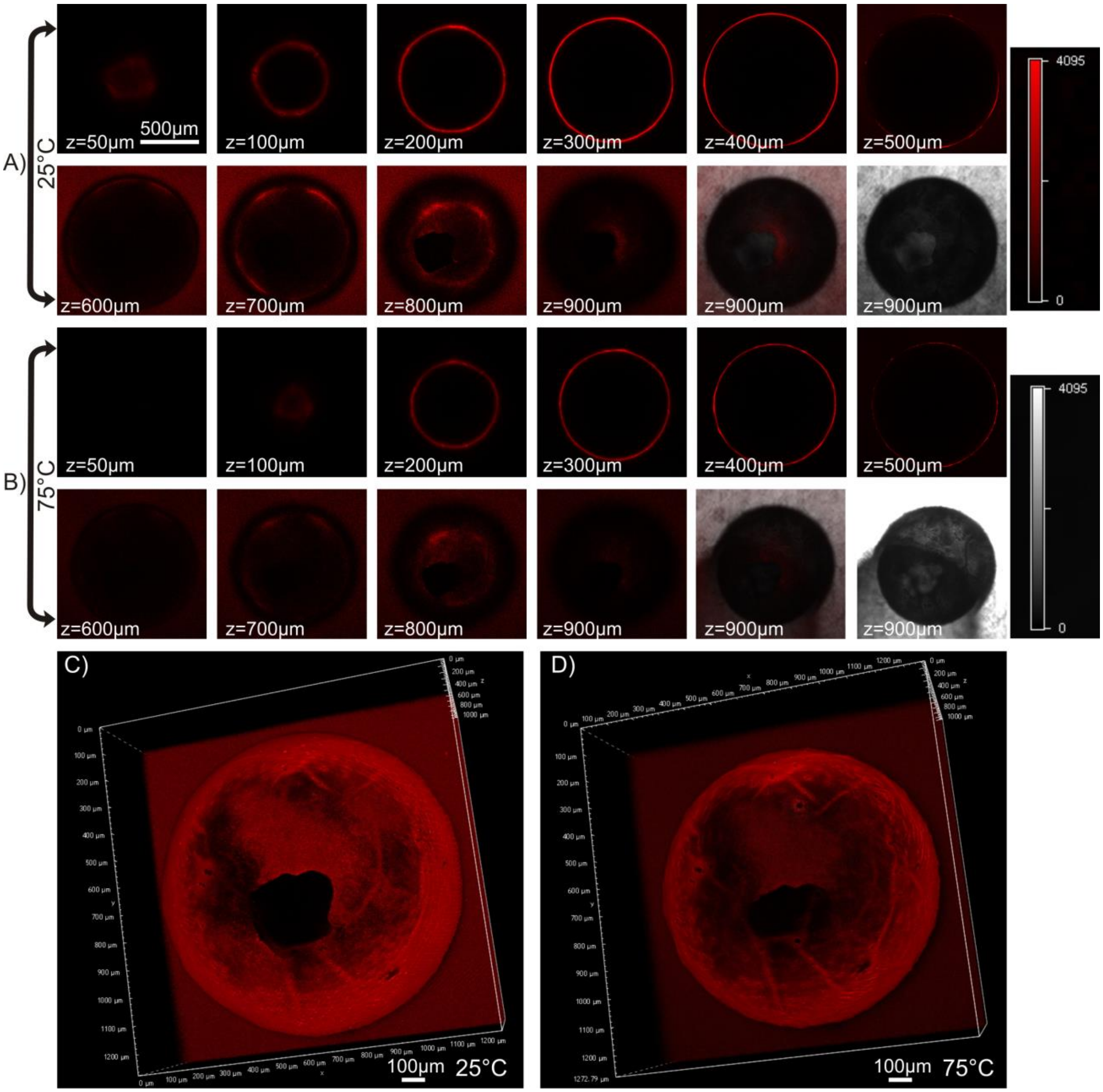

Fig. S6. Fluorescence confocal microscopy images of a shell with an opening. (**A**) Sequential *z*-scans obtained at 25°C. (**B**) Snapshots of confocal *z*-scans obtained at 75°C similar to A. (**C** and **D**) Full 3D images of the shell taken at 25°C and 75°C, respectively, show a substantial decrease in shell size.

A slightly irregular spherical cap fragment was cut from the shell shown in fig. S4 and placed on a small glycerol bath in the hot stage of the microscope, concave side up, for study of its actuation behavior, see fig. S7 and Movie S3. Other fragments are shown in Movie S5, discussed in the main paper. In fig. S7 we see a (fully reversible) shape change in the temperature range 60-70°C where $|\langle P_2 \rangle|$ diminishes towards zero. As expected, the surface area contracts but here the interfacial tension with the glycerol bed also pulls the fragment down, resulting in a shape

change from spherical cap towards cylindrical coil. Since the cut was manual, the fragment perimeter is not a perfect circle, breaking the symmetry, which is probably the reason that the direction of flattening, and the perpendicular direction of coiling, are the same in every repetition. Along the former direction (horizontal in the figure) the LCE may appear to have expanded somewhat, but this is an illusion due to the flattening. Interestingly, the reversible spherical cap–cylindrical coil shape change transports a net amount of the surrounding liquid towards the fragment center, hence the LCE is doing work during an actuation cycle.

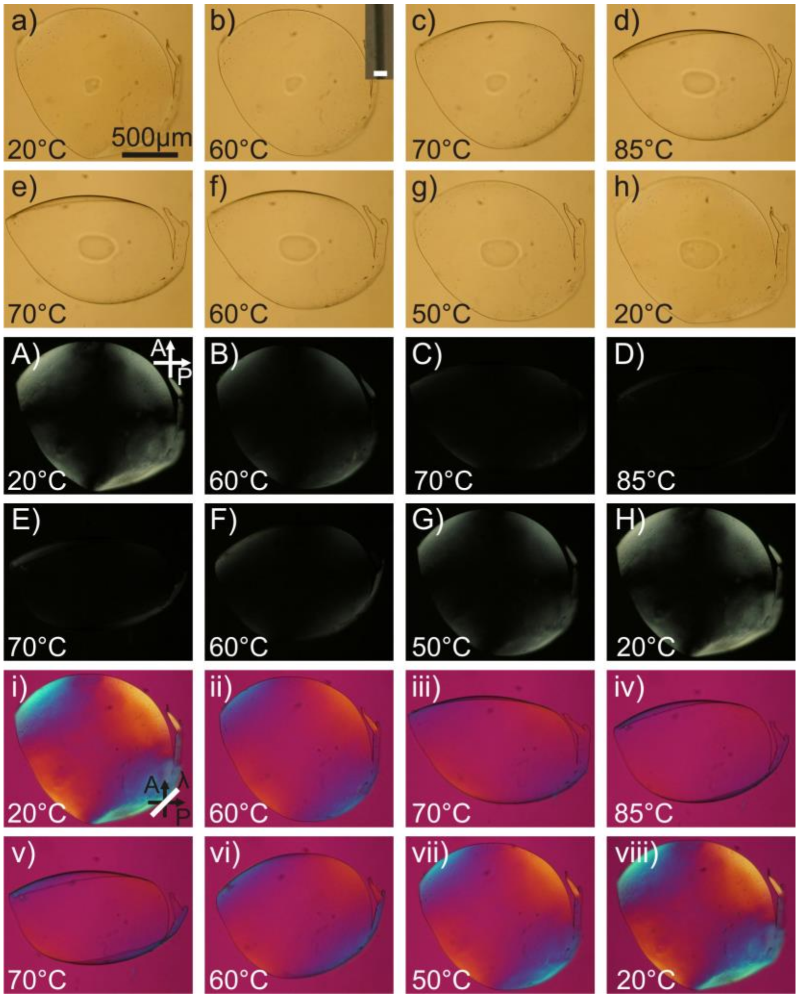

Fig. S7. Transmission POM investigation of thermal response of $\langle P_2 \rangle < 0$ LCE shell fragment. The fragment was cut from the shell in fig. S4. The fragment responds strongly with change in both shape and Δn. (a-h) Imaging without analyzer, (**A** to **H**) between crossed polarizers, and (i-viii) between crossed polarizers with first-order λ plate inserted (optic axis from lower left to upper right). The inset in (b) shows the shell thickness of 4.7 µm which was taken with a high magnification objective.

In panels C-E and iii-v we observe a weak remnant birefringence at the top left and bottom right parts of the fragment, where it coils up maximally in the third dimension. This reveals that, although the order parameter increases from its initial $\langle P_2 \rangle < 0$ value, it does not fully reach $\langle P_2 \rangle = 0$ at high temperatures. However, the offset is so small that the remnant birefringence can only be detected if the light path through the sample is greatly increased, as is the case in the points of maximum coiling.

Parallelly, we also studied the thermal response of dye-doped shell fragment. As shown in fig. S8, the shell fragment curls up into the third dimension compared to the 25°C state. Such a response indicates that the total surface area is decreasing upon heating. Since the dye is trapped in the network during the formation of LCE shell, it is not easy to determine the change in

thickness experimentally, as the fluorescent signal gets affected by the local LCE network distortions.

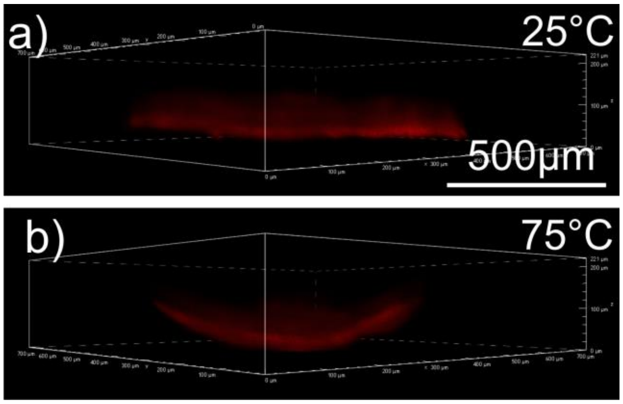

Fig. S8. Fluorescence confocal microscopy images of a shell fragment. (**a**) 3D view of the shell fragment at 25°C. (**b**) 3D view of the same fragment curling up into the z-direction at 75°C. The thermal response of the shell fragment indicates that it minimizes its surface upon heating, thus the shell thickness should be increased to conserved the volume of the LCE material.

Simulation of shell actuation and comparison with experiment

A phenomenological model was used to help illustrate the effects of a negative order parameter LCE shell during actuation. Negative order LCEs should demonstrate actuation behavior opposite that of traditional monodomain LCEs with $\langle P_2 \rangle > 0$. When cooled from the isotropic state to the nematic state, expansion should occur orthogonally to the director, **n**. Therefore, as negative order is established, the surface of the shell should try to expand, while the thickness of the shell in the radial direction should contract. This poses an interesting condition when the inside of the shell is filled with an incompressible fluid, such as water.

The main purpose of this simulation was to computationally evaluate the buckling/wrinkling phenomenon observed in the experiments on LCE shells photocrosslinked at high temperature, where the order parameter is negative but closer to zero than at room temperature. On cooling, the order parameter becomes increasingly negative, thus following the simulated scenario. Images of the simulated centered and offset spheres can be seen in fig S9. In the first condition of a perfectly spherical shell, no surface buckling was observed even with the maximum applied surface strain of 4%; however, the model showed periodic lines of stress across the shell surface. The magnitudes of these stress lines increased with increased amounts of strain. This periodic variation in stress could potentially induce periodic wrinkling across the entire shell surface. While worthy of further exploration, this behavior is difficult to achieve experimentally due to the manufacturing process.

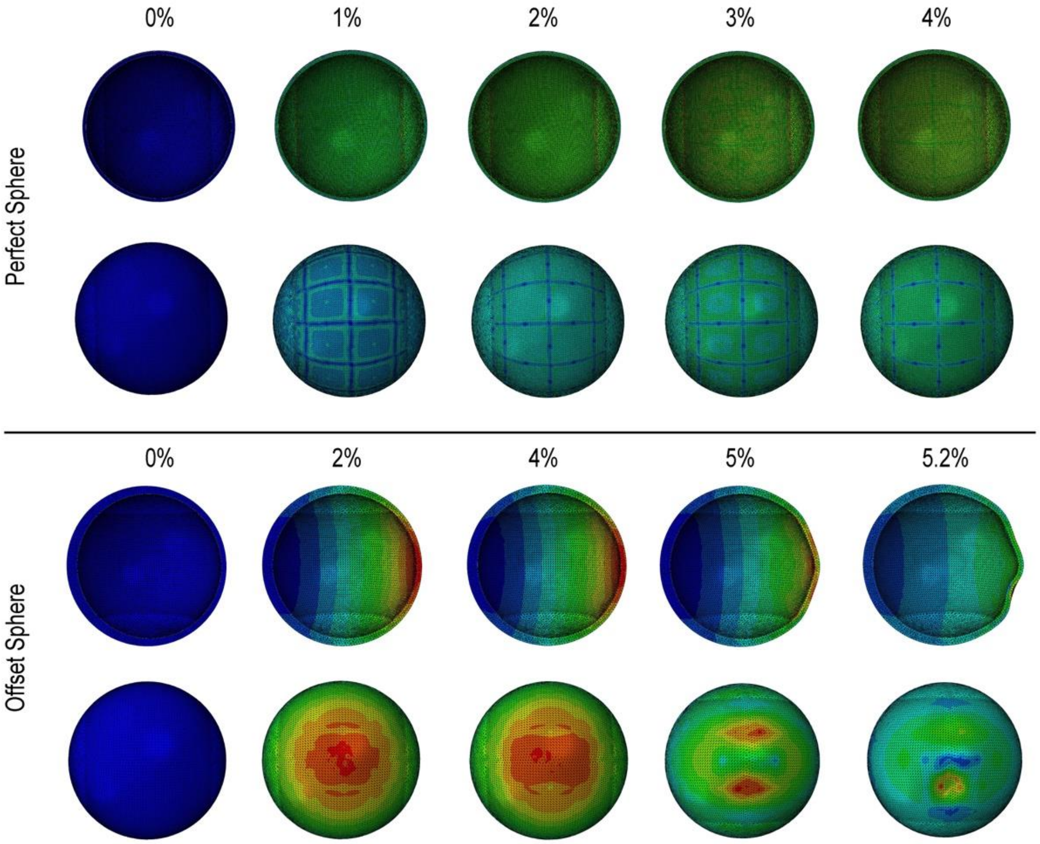

Fig. S9. LCE shells were modeled using ABAQUS finite element software. The perfectly centered shell model (top) displayed periodic lines of stress across the surface which could potentially induce wrinkling seen experimentally. The offset model (bottom) developed wrinkling at the thinnest portion of the shell. Stress initially concentrates circumferentially around the thinnest wall portion as seen above, and then separates into multiple concentrated areas at approximately 5% induced strain. For each case the shell is visualized along two different directions and as cross section (upper half) and as complete sphere (lower half), respectively.

The actual LCE shells can be asymmetric because they are made under the influence of gravity, using liquids with different densities. This creates shells with an offset inner cavity. The shells thus have varying thickness around the surface, with one thickest and one thinnest point. Simulations with offset inner sphere therefore match the experimentally produced shells better. In the simulation, this shell type displayed a unique behavior in its stress concentration along the thinnest portion of the shell while the rest of the shell remained relatively uniform in stress. Stress tended to increase linearly as one approaches the thinner wall. Maximum stresses seen in this model were higher than those seen in the centered sphere model. As it was not computationally efficient to continue past 4% strain in the perfectly centered sphere, the induced strain was limited to 4%; however, the offset sphere was able to continue to 5.2% induced planar strain at which point a buckling in the shell surface appeared near the thinnest wall similar to what was seen experimentally.

The buckling seen at the thinnest portion of the shell wall in the simulations with offset inner sphere can be explained by the shell trying to expand in diameter but being constrained by the incompressibility of the fluid inside. Therefore, to satisfy an increase in surface area of the shell wall but maintain a constant inner volume, buckling must occur to break the symmetry of the shell. In this case, buckling will begin at the thinnest portion of the shell wall.
The behavior observed in the offset shell model is more realistic as the stress tends to concentrate around non-uniformities and form wrinkles in areas with thinned walls. It should be noted that this model was an idealized approach to analyze the wrinkling phenomenon seen in the LCE shells. There are many factors, which could affect the behavior of the shells. Additionally, the induced strains applied in this model were computationally limited to 5.2% or less; however, the behavior seen in the models indicates that the isovolumetric conditions within the LCE shell can induce buckling/wrinkling behavior as seen experimentally. Areas in the shell which experience higher stresses will tend to buckle inward due to the incompressibility of the fluid within the shell.

Interestingly, the shape change observed in the corresponding experiment (see discussion in main text) appears to have a memory, as every cooling cycle generates the same appearance, indicating buckling at identical points and collapsing along identical paths every time (see Movie S4). The simulation results suggest that the locations of buckling are set by local variations in shell thickness, arising due to density mismatch of LCE precursor mixture and the inner aqueous phase during the osmosis stage (*31*).

Twist and bend deformation in a ribbon-shaped shell fragment
The shell is cut using a scalpel in a frozen state. The slicing of the shell along the equator results in a self-closing ribbon shaped fragment. At 55°C, this fragment spans about 600 µm in the horizontal direction whereas it is about 390 µm vertically. Upon heating to 85°C the ribbon shrinks in the horizontal direction to about 520 µm, equivalent to a 13% reduction. In the vertical direction the ribbon at 85°C measures 440 µm, equivalent to a 13% expansion compared to the 55°C state, thus corresponding well with the shrinkage in the horizontal direction. From the onset, the ribbon, as it is floating on the glycerol surface, exhibits a back-and-forth twisting, with the upper edge (closer to the camera) leaning inwards along the top and bottom, whereas it leans outwards along the left and right. The actuation involves a reversible twist-bend deformation that emphasizes this shape, the left, right, upper and lower part of the ribbon being flatter at high temperature, whereas the mediating sections exhibit stronger twist and bend, see movie S5.

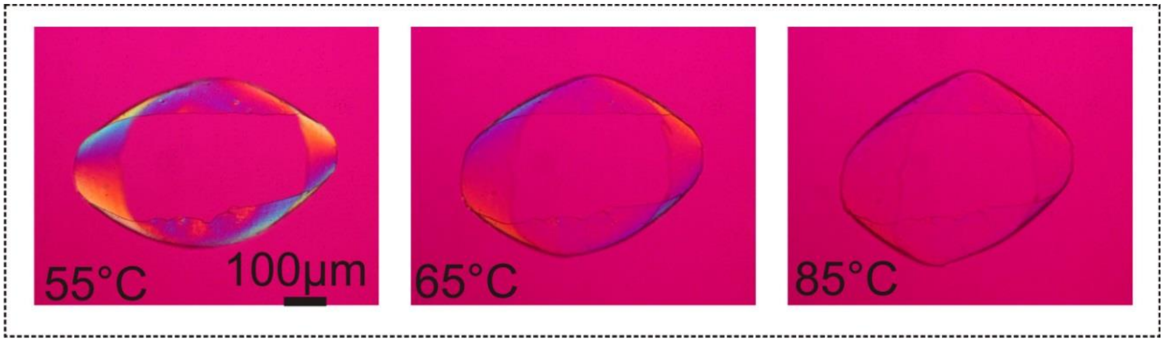

Fig. S10. LCE shell fragment in the shape of twisted ribbon. The snapshots during the actuation cycle show 13% reduction in its width and the same amount of increase in its height, together with an increased twist-bend deformation in four points.

Macroscopic LCE with negative mesogen order parameter

The transferability of the concept was demonstrated in non-shell-shaped, and macroscopic LCEs through mechanical uniaxial compression between the first and second stage reactions. These samples were prepared by initially creating a cylindrical LCE with 15% excess acrylate. The 5mm tall cylinder was compressed along the disk symmetry axis between glass slides with clamps until the cylinder height was a uniform 2mm, see fig. S11a. The compression assembly was placed in a UV oven for 30 minutes on each side to ensure complete crosslinking of the excess acrylate within the LCE structure, locking in place the negative order mesogen arrangement. The optical properties of this thick disk (fig. S11b) are unfortunately not uniform enough for proper optical characterization, but the mechanical actuation (main manuscript) demonstrates that the negative order parameter ground state was achieved.

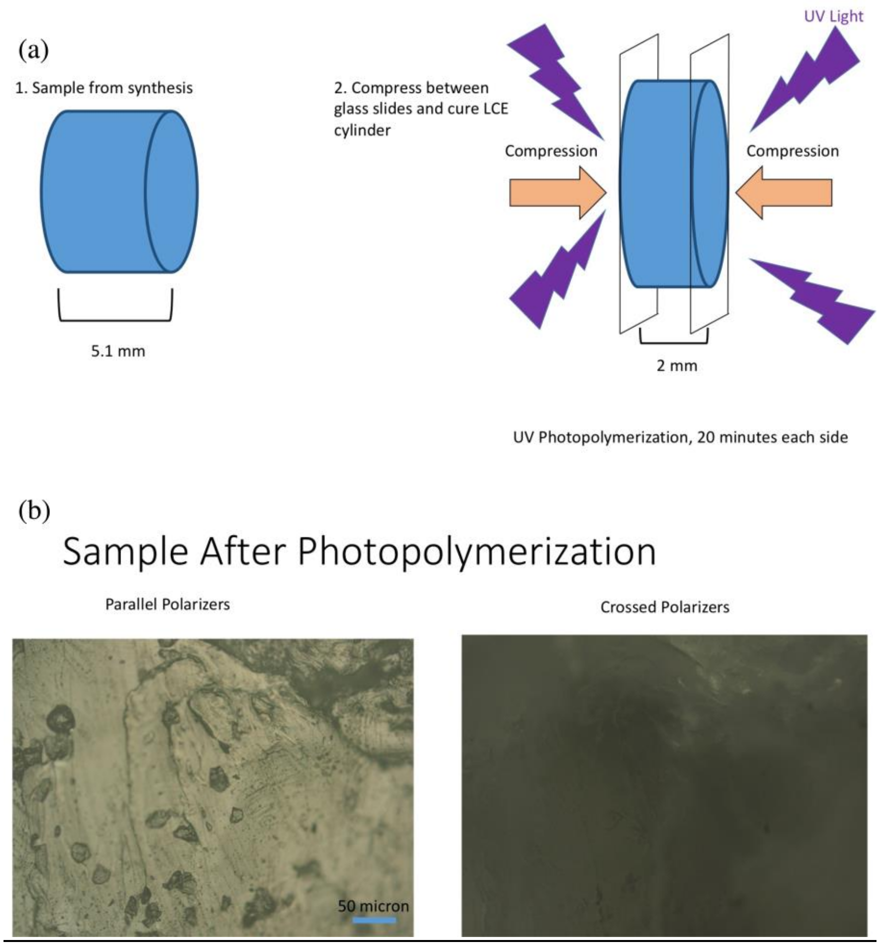

Fig. S11. Macroscopic disk with negative mesogen order parameter. (**a**) Schematic illustration of the manufacturing procedure. (**b**) Texture observed in the optical microscope (reflection) with parallel (left) and crossed (right) polarizers.

Movie S1. The video shows thermal actuation cycles (25° to 75°C) of a pristine shell UV cross-linked at 35°C, recorded with a first-order waveplate, crossed polarizers, and one polarizer in the light path, respectively. The experiment was carried out in a rectangular glass capillary containing pure water.

Movie S2. Thermal actuation of a shell UV cross-linked at 35°C with a hole at the top, immersed in glycerol. The movie was taken with a first order waveplate, crossed polarizers and one polarizer in the light path respectively.The experiment was carried out in a rectangular glass capillary containing glycerol.

Movie S3. The video shows actuation of a fragment cut from a shell UV cross-linked at 35°C. The observation is with a first order waveplate, crossed polarizers and one polarizer in the light path respectively. The experiment was carried out on a glass plate where the shell fragment is floating on glycerol. In the last part one can see how the fragment collects glycerol to the center during an actuation cycle.

Movie S4. Thermal actuation of a shell UV cross-linked at 60°C. The movie was taken with a first order waveplate, crossed polarizers and one polarizer in the light path respectively. The experiment was carried out in a rectangular glass capillary containing pure water.

Movie S5. Complex modes of actuation observed by cutting a shell UV cross-linked at 35°C into different topological objects such as a cap shape, a self-closed ribbon, and a long spiral stripe shape. The experiment was carried on a glycerol bath with a first order waveplate inserted into the light path, between the crossed polarizers.
